# Supplementary material for: G-quadruplexes originating from evolutionary conserved L1 elements interfere with neuronal gene expression in Alzheimer’s disease
Source: Nat Commun. 2021 Mar 23;12:1828. doi: 10.1038/s41467-021-22129-9 (PMC7987966; doi:10.1038/s41467-021-22129-9)
Supplement: Supplementary file 1 — Supplementary Information [file 41467_2021_22129_MOESM1_ESM.pdf]

Supplementary Information

G-quadruplexes originating from evolutionary conserved L1 elements interfere with neuronal gene expression in Alzheimer's disease

Roy Hanna[1], Anthony Flamier[1,3], Andrea Barabino[1], and Gilbert Bernier[1,2]

[1] Stem Cell and Developmental Biology Laboratory, Hôpital Maisonneuve-Rosemont, 5415 Boul. l'Assomption, Montreal, QC, Canada, H1T 2M4

[2] Department of Neurosciences, University of Montreal, Montreal, QC, Canada

[3] Present address: Whitehead institute of biomedical research, 455 Main Street, Cambridge, MA 02142

* Running title**:** *G-quadruplexes in Alzheimer's disease*

Correspondence: gbernier.hmr@ssss.gouv.qc.ca

Content:
14 supplementary Figures

Hanna et al. 2020

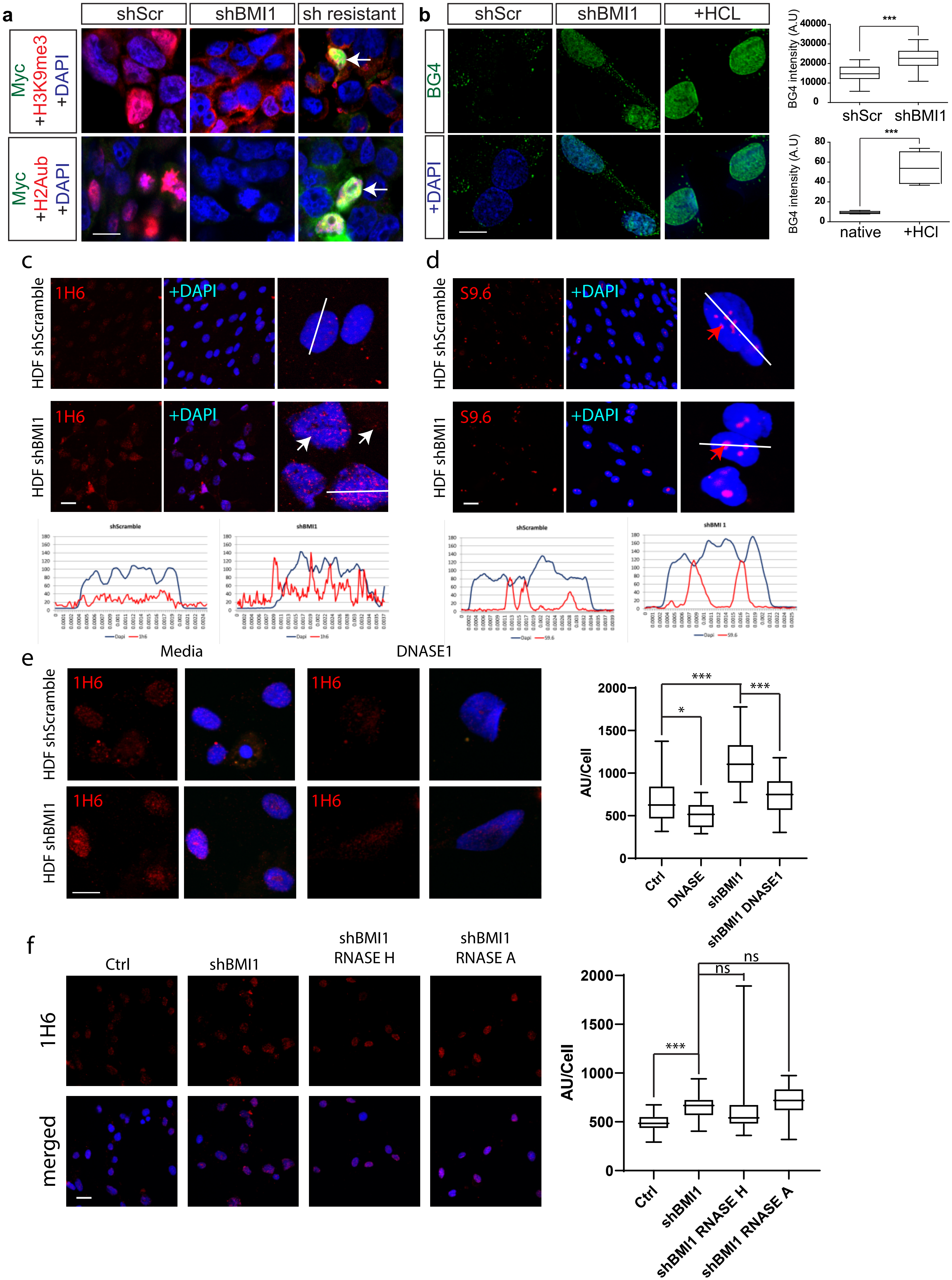

Supplementary Figure 1

Supplementary Figure 1. Validating the 1H6 antibody as a suitable tool to detect G4 structures

a. HCA2 cells were infected with the shScramble or shBMI1 viruses together with a BMI1myc DNA construct that was shRNA-resistant. 72 hours later, paraformaldehyde-fixed cells were immunolabeled and counterstained with DAPI. Cells positive for the BMI1myc construct are indicated (white arrow). Note the rescue of H2Aub and H3K9me3 in shBMI1 cells also expressing the shRNA-resistant BMI1myc construct. Scale bar: 15µm.

b. Formaldehyde fixed HCA2 cells infected with shScramble or shBMI1 viruses, or denaturated using 3M of HCl, were immunolabeled and counterstained with DAPI and BG4 antibody. These antibodies were used to detect G-quadruplexes structures. The graphs show the quantification of BG4 signal intensity in each cell with the relevant unpaired T-test with two tails. $P \leq 0.05$*, ≤ 0.01**, ≤ 0.001***. The box plots show the minima and the maxima values delimited by the whisper, the box delimites the 25th percentile and 75th percentile, and the line inside the box define the mean. A total of 100 cells were quantified in each condition. Scale Bar: 10 µm.

c. Immunofluorescence analysis of Human Dermal Fibroblast (HDF) infected with shScramble or shBMI1 and stained with 1H6. A zoom of the merged image is presented with a white line that was used to draw the profile plot presented under the images. Where we can see the intensity profile of the two markers. Scale Bar: 10 µm.

d. Immunofluorescence analysis of Human Dermal Fibroblast (HDF) infected with shScramble or shBMI1 and stained with S9.6 and antibody that recognizes the R-Loops. A zoom of the merged image is presented with a white line that was used to draw the profile plot presented under the images. Where we can see the intensity profile of the two markers. We can notice that the S9.6 peaks are exclusively in the nucleoli of the cells colocalizing with the valleys in the DAPI profile. Scale Bar: 10 µm.

e. Immunofluorescence analysis of Human Dermal Fibroblast (HDF) infected with shScramble or shBMI1 and stained with 1H6. In order to confirm that the foci detected by the 1H6 antibody were G4 DNA structures, we treated the cells with DNASE1 that rescued the phenotype. The quantification of the signal per cell is given in the box and whiskers plot highlighting the statistical significance. Statistical differences were analyzed using unpaired T-test with two tails $P<0.05$ *,

P<0.001 ***. The box plots show the minima and the maxima values delimited by the whisper, the box delimites the 25th percentile and 75th percentile, and the line inside the box define the mean. A total of 100 cells were quantified in each condition. Scale Bar: 10 µm.

f. Immunofluorescence analysis of Human Dermal Fibroblast (HDF) infected with shScramble or shBMI1 and stained with 1H6. In order to confirm that the foci detected by the 1H6 antibody were G4 DNA structures we treated the cells with RNASE H or RNASE A, both failed to rescue the phenotype. The quantification of the signal per cell is given in the box and whiskers plot highlighting the statistical significance. Statistical differences were analyzed using unpaired T-test with two tails $P<0.05$ *, $P<0.001$ ***. The box plots show the minima and the maxima values delimited by the whisper, the box delimites the 25th percentile and 75th percentile, and the line inside the box define the mean. A total of 100 cells were quantified in each condition. Scale Bar: 10 µm.

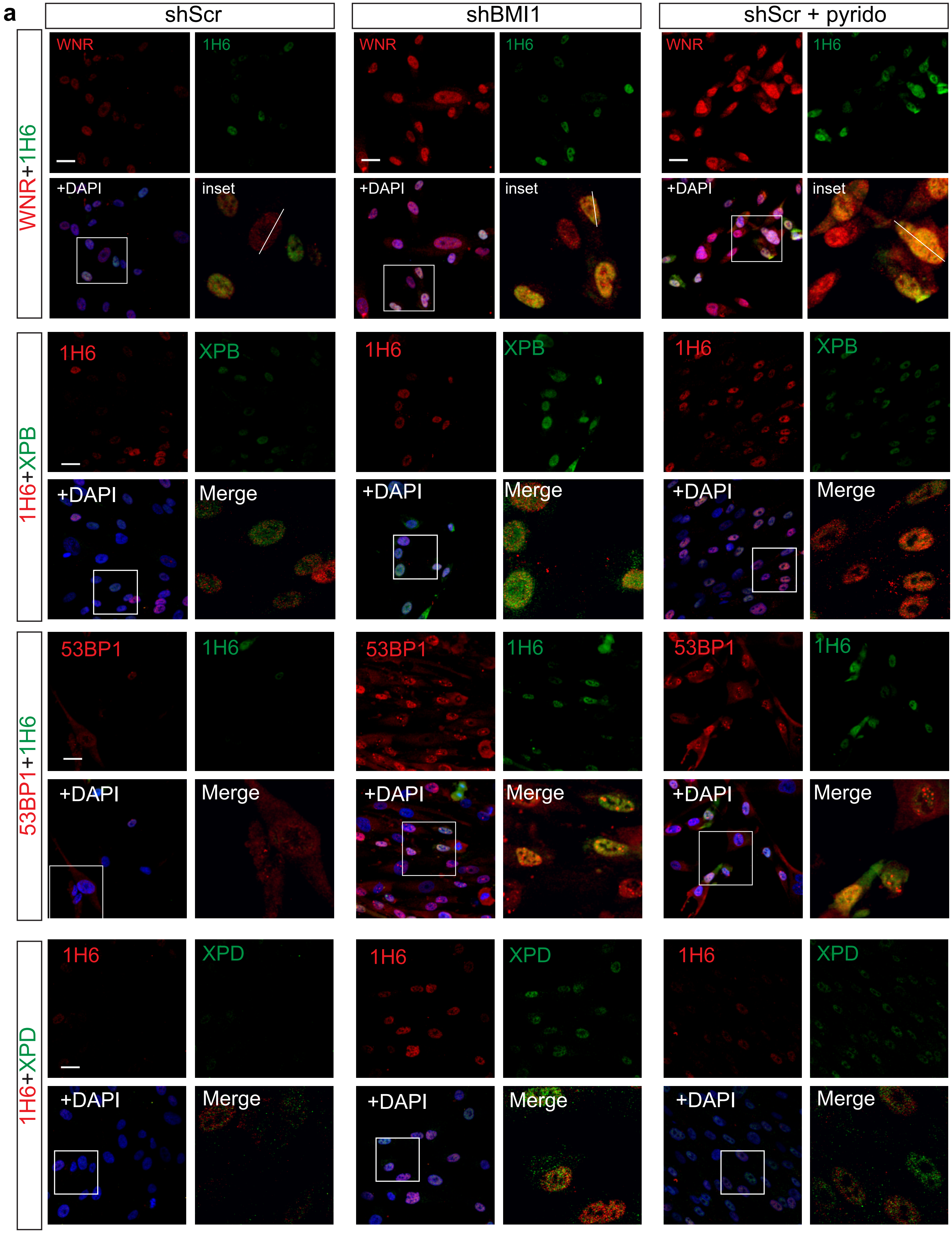

Supplementary Figure 2

Supplementary Figure 2. The 1H6 antibody show co-localization with DNA helicases upon BMI1 knockdown in human dermal fibroblasts

a. Formaldehyde fixed HCA2 cells were co-immunolabeled with 1H6 and antibodies against various helicases, counterstained with DAPI. Crop with higher magnification of the area is indicated by the square. Scale Bar: 10 µm.

Hanna et al. 2019

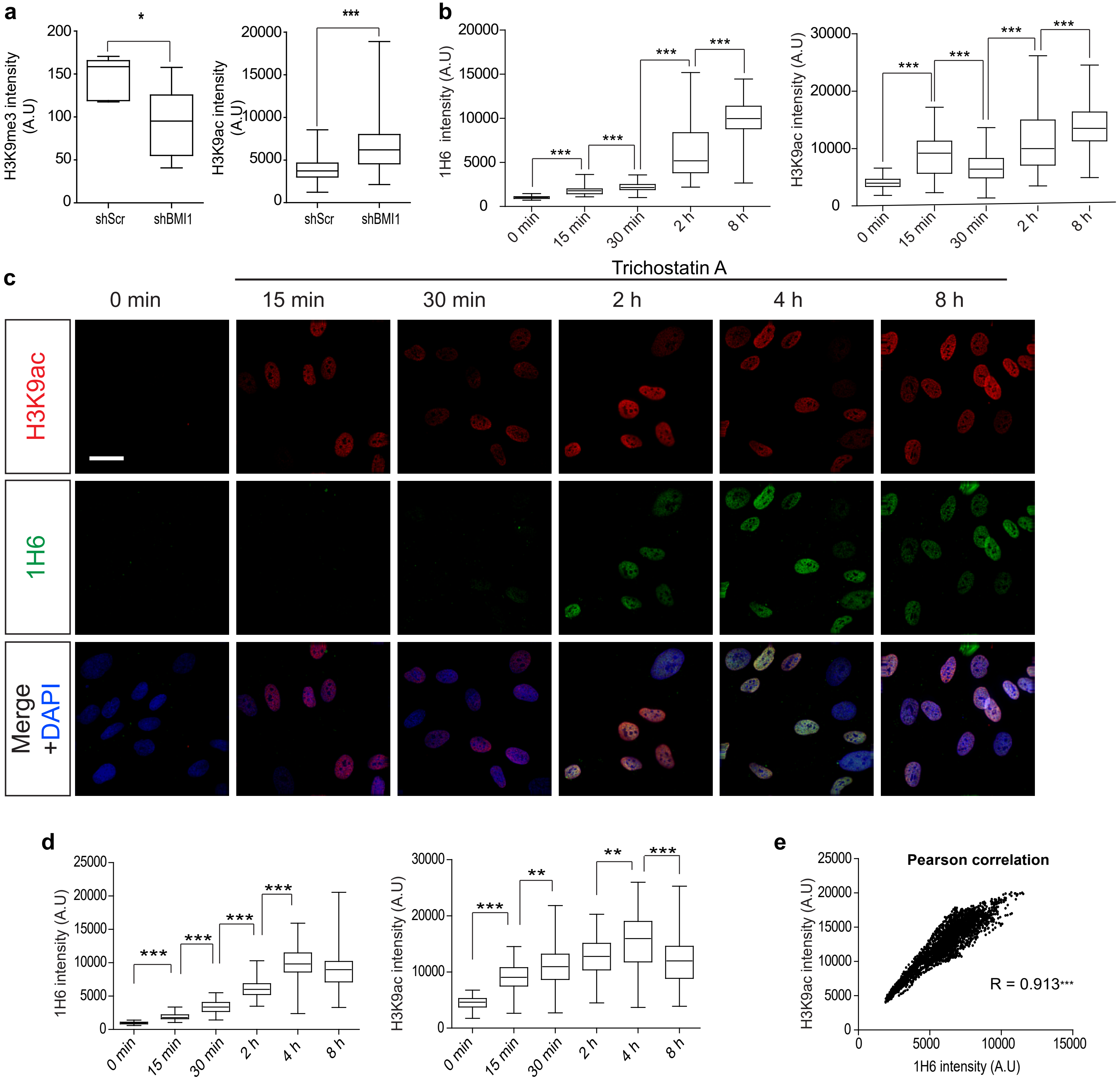

Supplementary Figure 3

Supplementary Figure 3. Chromatin relaxation induces the formation of G4 structures

a. Quantification of the $H3K9^{me3}$ and the $H3K9^{ac}$ signal from Figure 1I. BMI1 knockdown resulted in decreased $H3K9^{me3}$ level and increased $H3K9^{ac}$ level. Statistical differences were analyzed using unpaired T-test with two tails. The box plots show the minima and the maxima values delimited by the whisper, the box delimites the 25th percentile and 75th percentile, and the line inside the box define the mean. $P<0.05$ *, $P<0.001$ ***. A total of 150 cells were quantified in each condition.

b. Quantification of the $H3K9^{ac}$ and 1H6 nuclear signal in the cells that were treated with sodium butyrate in Figure 1J. Statistical differences were analyzed using unpaired T-test with two tails. $P<0.05$ *, $P<0.001$ ***. The box plots show the minima and the maxima values delimited by the whisper, the box delimites the 25th percentile and 75th percentile, and the line inside the box define the mean. A total of 150 cells were quantified in each condition.

c. Immunofluorescence pictures of HCA2 cells treated with 5ng/mL of trichostatin A for the time indicated. Scale bar: 35 μm.

d. Mean signal intensity of 1H6 and $H3K9^{ac}$ in each nucleus were measured and plotted in a box and whisker graph. Statistical differences were analyzed using unpaired T-test with two tails. $P<0.05$ *, $P<0.001$ ***. The box plots show the minima and the maxima values delimited by the whisper, the box delimites the 25th percentile and 75th percentile, and the line inside the box define the mean. A total of 300 cells were quantified in each condition.

e. Pearson correlation study of the coexpression between 1H6 and $H3K9^{ac}$ at the 2h time point plotted in a scatter graph with the Pearson coefficient indicated on the graph. $P \leq 0.05$*, ≤ 0.01**, ≤ 0.001***. The box plots show the minima and the maxima values delimited by the whisper, the box delimites the 25th percentile and 75th percentile, and the line inside the box define the mean. A total of 300 cells were quantified in each condition.

Hanna et al. 2019

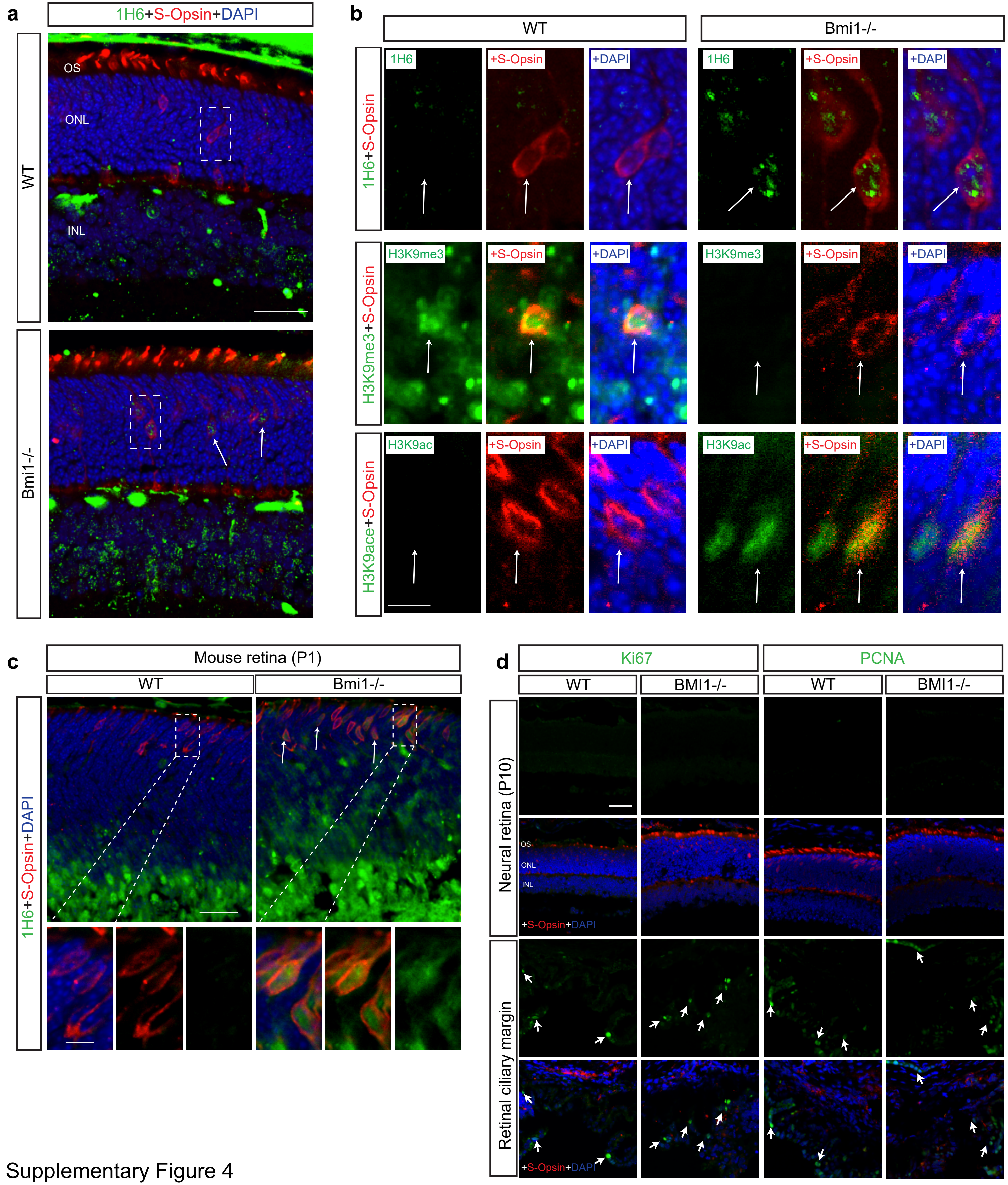

Supplementary Figure 4

Supplementary Figure 4. ***Bmi1*** **deficiency leads to the formation of G4 structures in mouse photoreceptors**

a. IF analyses on WT and *Bmi1*$^{-/-}$ mouse retinal sections at P10 using the 1H6 and anti-S-Opsin antibodies. Crops with higher magnification of the area indicated by the respective dashed rectangles are presented in Figure 1.e. Scale bars: 40µm.

b. IF analyses on WT and *Bmi1*$^{-/-}$ mouse retinal sections at P10 using 1H6, anti-H3K9^{ac}, anti-H3K9^{me3}, and anti-S-Opsin antibodies. Photoreceptors were labeled with H3K9^{ac} or H3K9^{me3} antibodies (white arrows). Scale bars: 12µm.

c. IF analyses of WT and *Bmi*1$^{-/-}$ mouse retinas at P1 using 1H6 (G4) and anti-S-Opsin antibodies. Crop with higher magnification of the area indicated by the respective dashed rectangles (at the bottom). S-cone photoreceptors with the induction of G4 structures are shown (white arrows). Scale bars: 40µm (top); 12µm (at the bottom).

d. IF analyses of WT and *Bmi*1-/- mouse retinas at P10 using the cell cycle markers anti-Ki67 and anti-PCNA and anti-S-Opsin (s-cone photoreceptors marker) antibodies. Positive control staining: positive cells for Ki67 and PCNA staining in the retinal ciliary margin (white arrows). Scale bars: 40µm.

Hanna et al. 2020

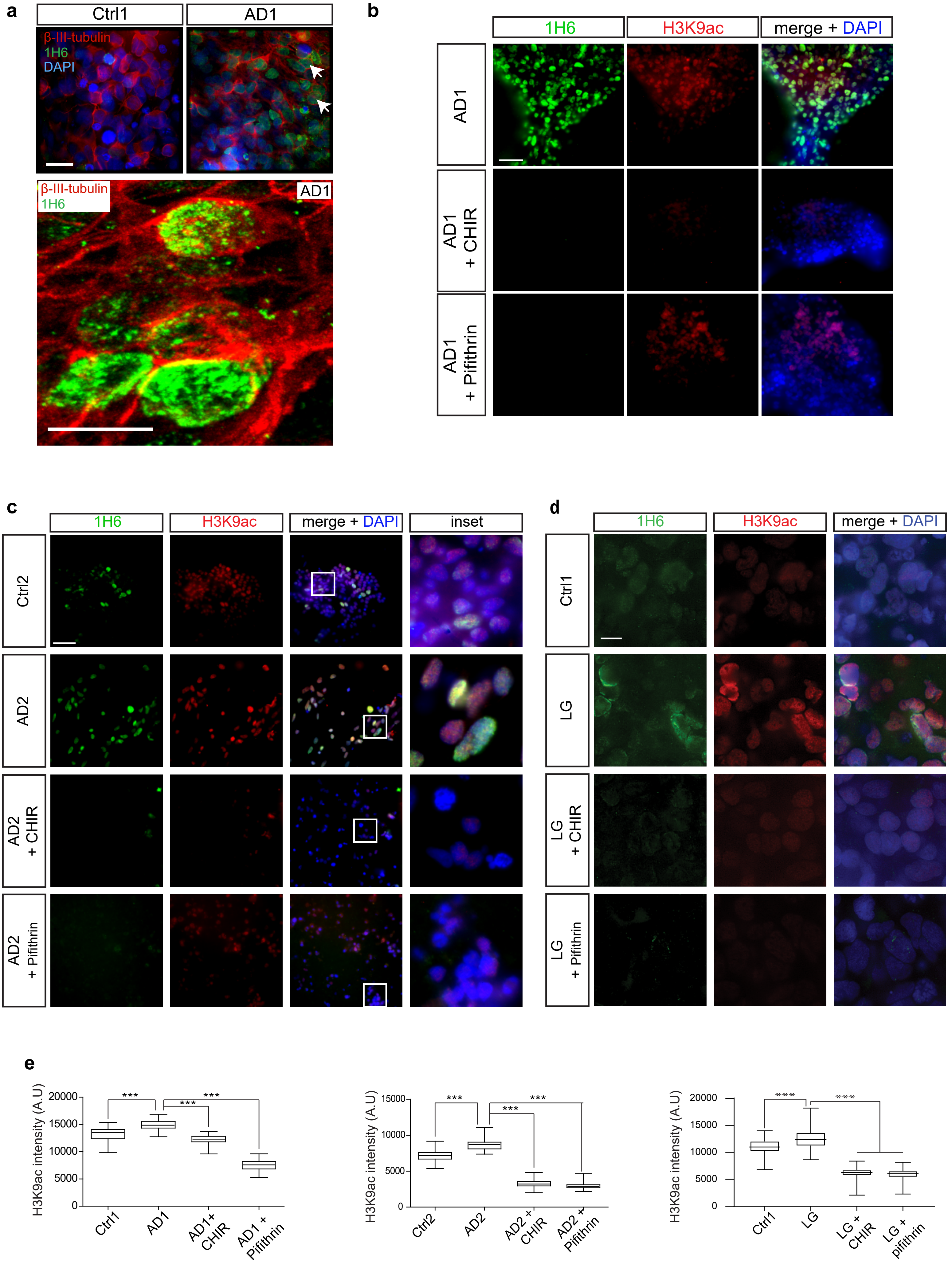

Supplementary Figure 5

Supplementary Figure 5. Formation of G4 structures in AD neurons can be reversed by re-establishing chromatin compaction

a. IF analysis showing that 1H6-positive cells in AD cultures are βIII-tubulin-positive neurons. Scale bar: 35 µm.

b. IF analyses of iPSC-derived control (Ctrl) and AD1 cortical neurons. Mature neurons were treated for 24h with an inhibitor of GSK3β (CHIR99021) or an inhibitor for p53 (pifithrin), then labeled with antibodies against 1H6 and $H3K9^{ac}$. Scale bar: 34 µm.

c. IF analyses of iPSC-derived control (Ctrl) and AD2 cortical neurons. Mature neurons were treated for 24h with an inhibitor of GSK3β (CHIR99021) or an inhibitor for p53 (pifithrin), then labeled with antibodies against 1H6 and $H3K9^{ac}$. Scale bar: 34 µm.

d. IF analyses of iPSC-derived control (Ctrl) and AD2 cortical neurons. Mature neurons were treated for 24h with an inhibitor of GSK3β (CHIR99021) or an inhibitor for p53 (pifithrin), then labeled with antibodies against 1H6 and $H3K9^{ac}$. Scale bar: 5 µm.

e. Mean $H3K9^{ac}$ fluorescence intensity/cell was quantified from figs S5b-d and plotted in a whisker and box plot. Statistical differences were analyzed using an unpaired T-test with two tails. $P \leq 0.001$***. All values are means ± SEM.

Hanna et al. 2020

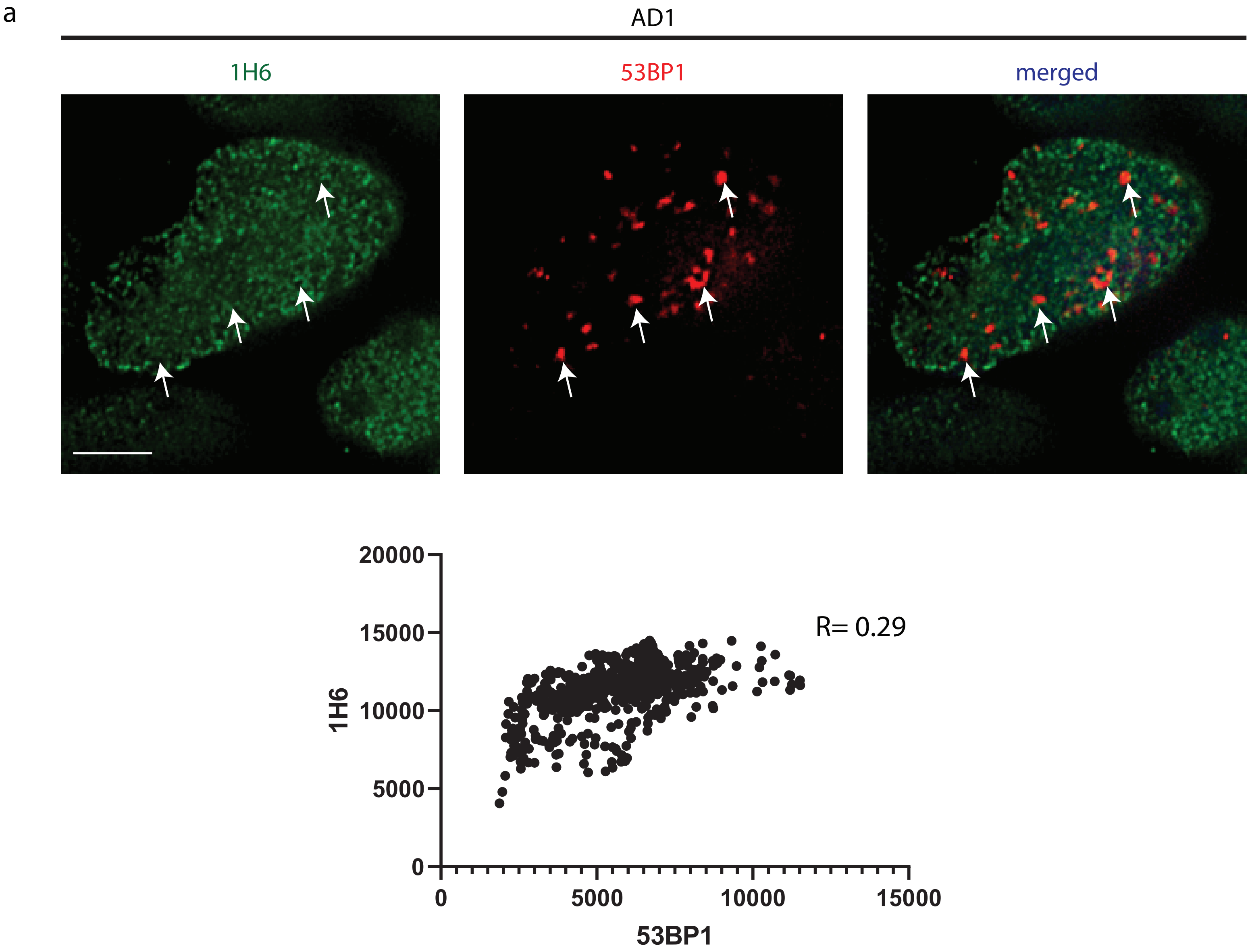

Supplementary Figure 6

Supplementary Figure 6. 53BP1 foci are not correlated with 1H6 foci in AD neurons

a. High magnification Immunofluorescence of neuronal AD1 nucleus stained with 1H6 and 53BP1. The white arrows indicate large 53BP1 foci location. The correlation between the 1H6 signal and the 53BP1 was calculated using Pearson correlation and plotted in the graph below, 30 cells were analyzed. The correlation coefficient was equal to R=0.29. Scale bar: 10 µm.

Hanna et al. 2020

a

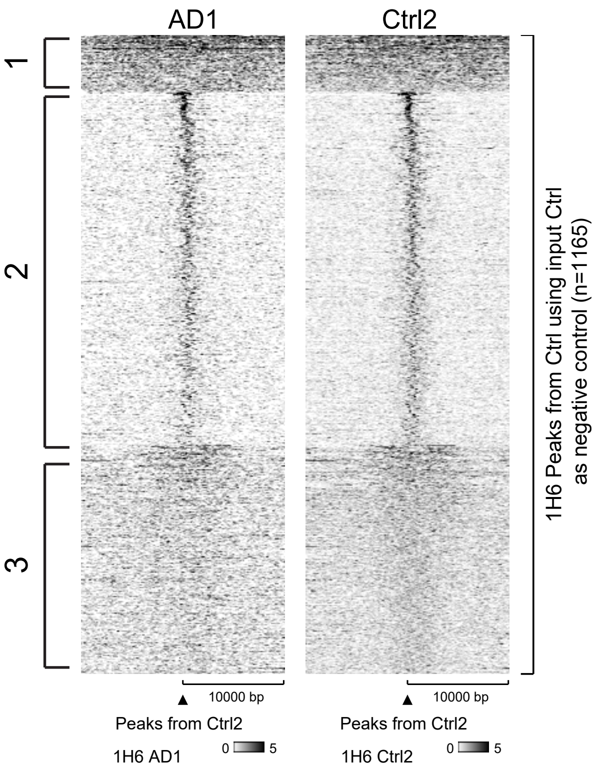

b

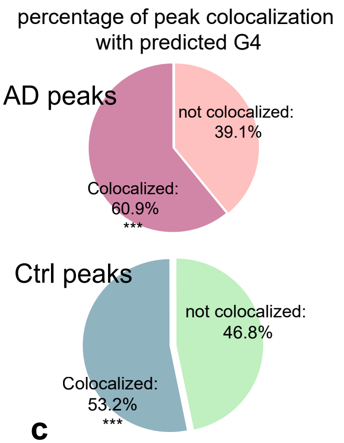

c

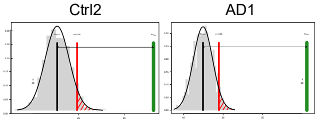

condition	pval	zscore
AD Vs G4	0.000999[illegible]	10.7358
Ctrl Vs G4	0.000999[illegible]	8.0749

d

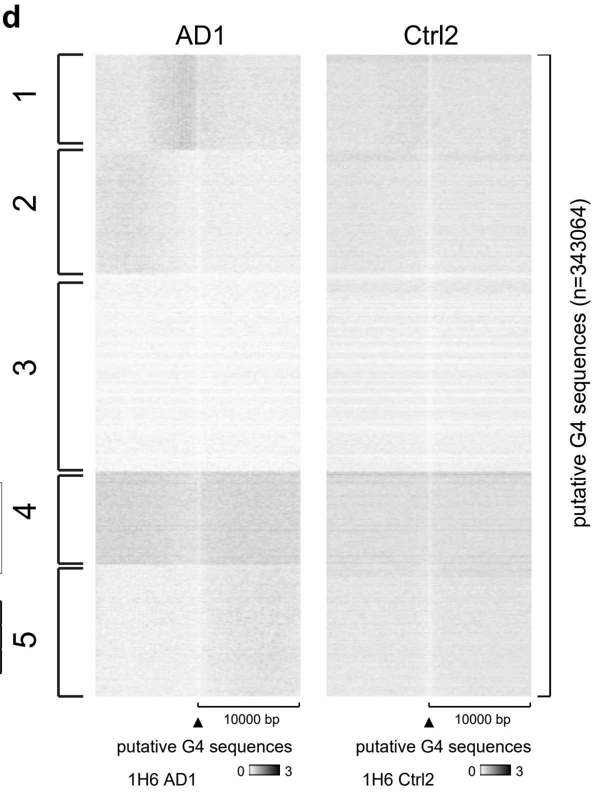

e

AD peaks	
	E value
colocalized with a putative G4	
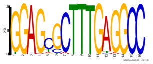	4.4e-2070
not colocalized with a putative G4	
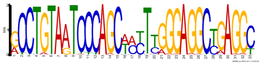	8.8e-342

Ctrl peaks	
	E value
colocalized with a putative G4	
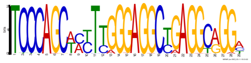	2.0e-689
not colocalized with a putative G4	
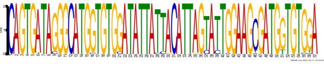	1.7e-713

f

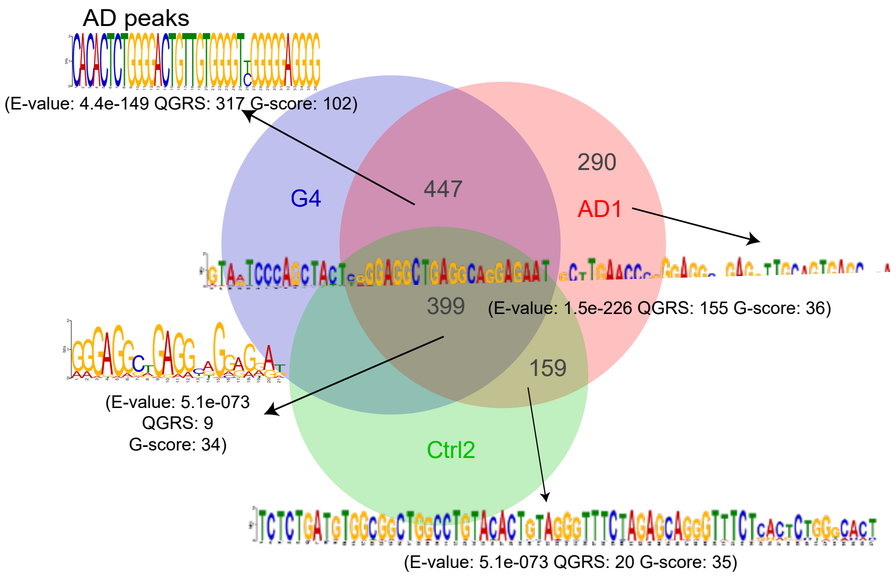

Supplementary Figure 7

Supplementary Figure 7. 1H6 peaks are more abundant in AD neurons and recognize a G4 motif

a. Heatmap of 1H6 ChIP-seq enrichment from AD1 neurons or control neurons (Ctrl2) centered on the Ctrl2 peaks with a +/- 10 Kbp. K-means clustering highlighted on the right was done using the Ctrl2 enrichment.

b. A pie chart showing the percentage of AD1 or Ctrl2 peaks that contain a G4 canonical sequence predicted by the Quadparser.

c. Permutation test on the colocalization between the 1H6 ChIP peaks and the G4 predicted sequences. A total of 1000 permutated sets of probes were randomly generated and annotated for G4 predicted sequences.

d. Heatmap of 1H6 ChIP-seq enrichment from AD1 neurons or control neurons (Ctrl2) centered on predicted G4 sequences +/- 10 Kbp. K-means clustering highlighted on the right was done using the AD enrichment.

e. MEME analyses of the peaks that colocalized or not wit a predicted G4 sequence, the corresponding E-value is shown for each motif.

f. Venn diagram showing the distribution of AD1 peaks in regards to containing or not: Ctrl2 peak or a predicted G4 sequence. For each category, a MEME analysis was performed, and a motif that can form a G4 structure was shown with the E-Value associated with them, the number of QGRS, and the highest G-score associated with these sequences.

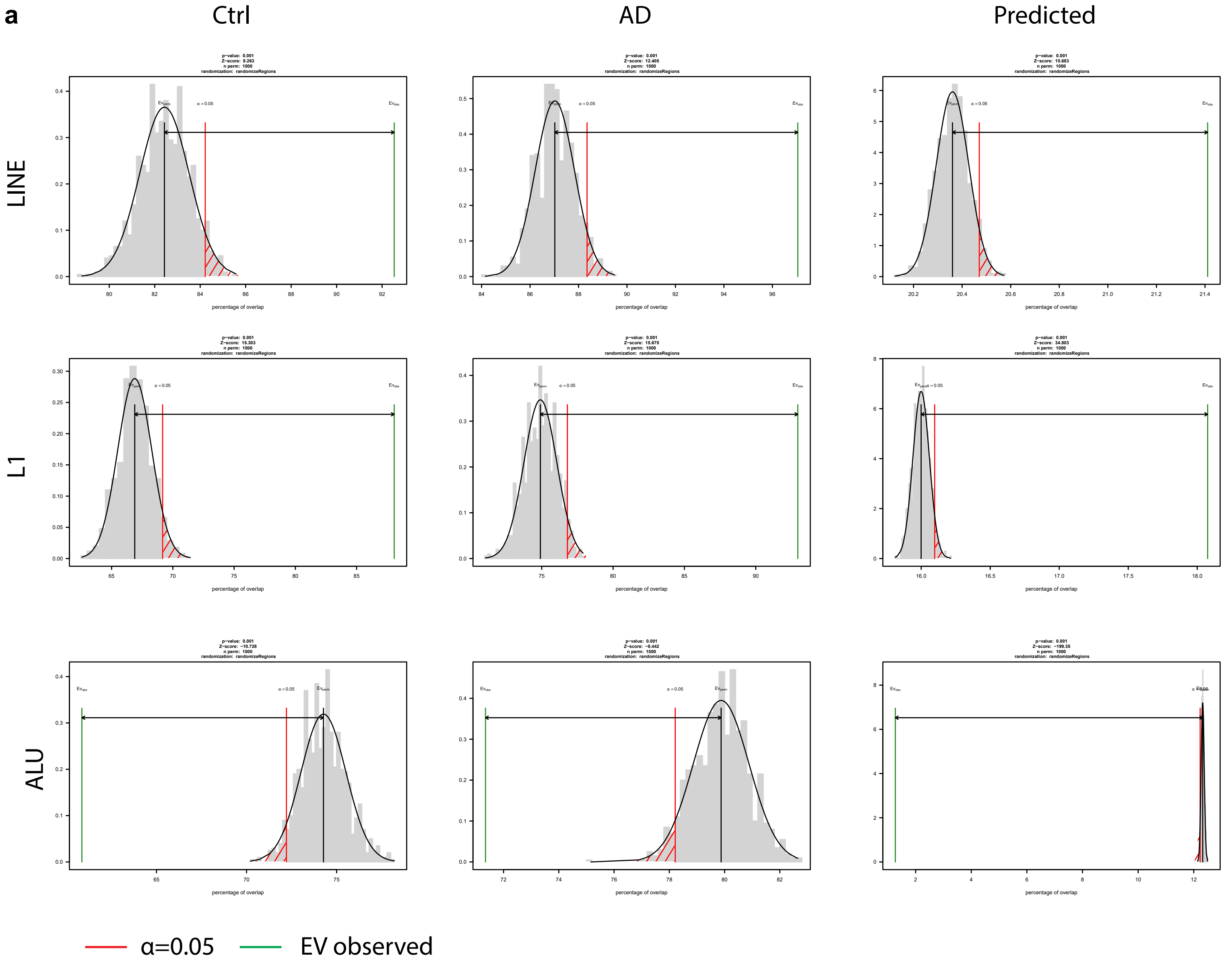

Supplementary Figure 8

Supplementary Figure 8. A sample of the raw data from permutations presented in Fig 4a.

a. Graphs showing the permutation tests of the most interesting families and subfamilies of repetition when compared to either the Ctrl peaks of 1H6, the AD peaks of 1H6 or the predicted G4 sequences in the genome. For each case 1000 set of random probes, that matched the specifications of the peaks tested, were generated (in this case Ctrl and AD 1H6 peaks, and predicted sequences) then compared to the repetition family (percentage of overlap). From these results we draw the gray collumnns showing how a random distribution would look like (black curve). The $\alpha = 0.05$ was then determined and plotted with a red line. On top of that the observed percentage of overlap was plotted with a green line and compared to the random distribution.

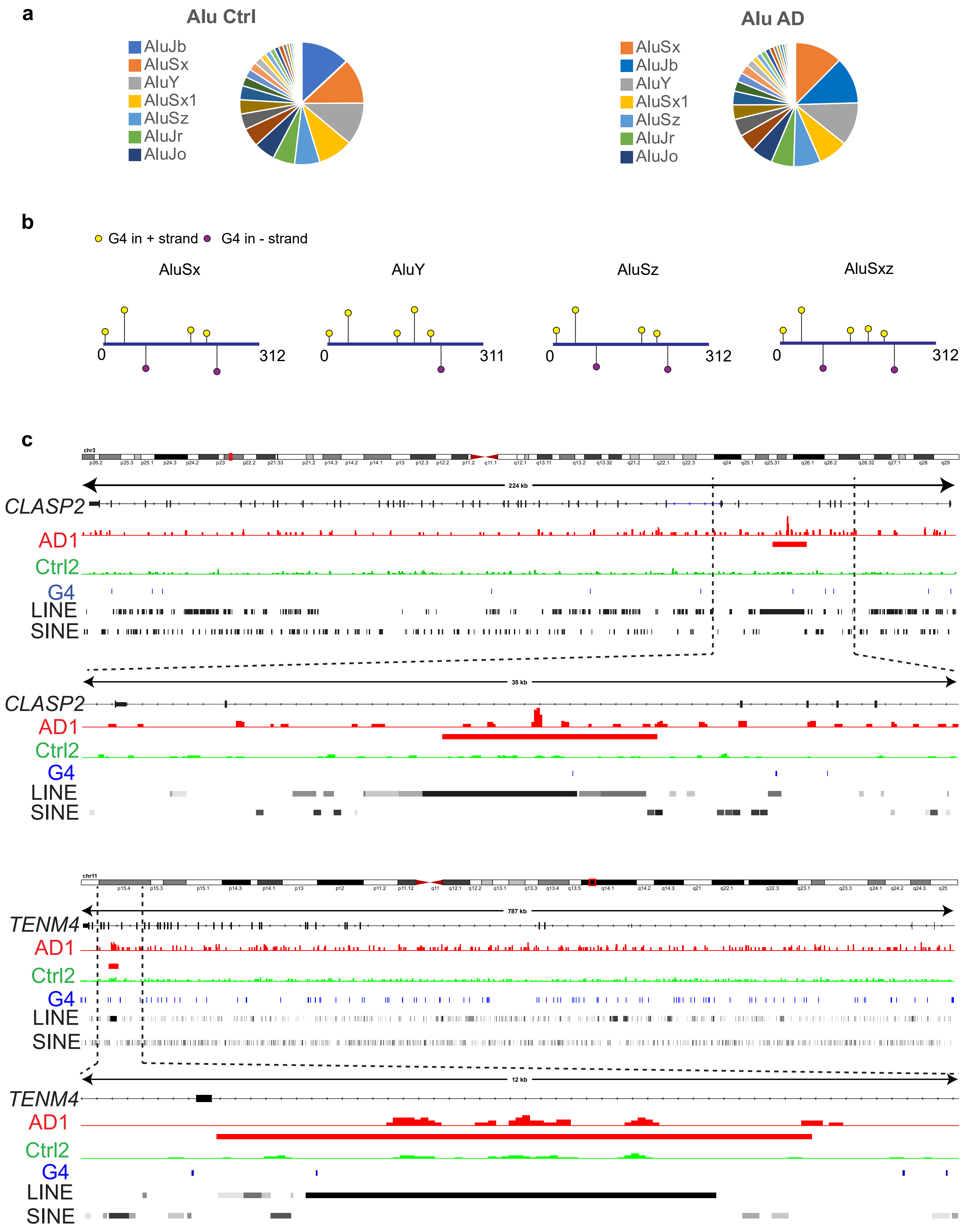

Supplementary Figure 9

Supplementary Figure 9. LINEs and SINEs are at the origin of G4 structures in human neurons

a. A pie chart showing the repartition of the repeats from ALU, the most represented SINE family in the AD1 and control (Ctrl2) peaks.

b. A visual representation showing the predicted G4 sequences on the consensus sequence of the repeats that are prevalent in the ALU family.

c. Physical maps showing enrichment of 1H6 ChIP-seq in two different gene bodies and the corresponding identified peaks. Along with the 1H6 enrichment, we plotted the repeat elements LINE and SINE with, the color of these repeats reflects their conservation with black being the most conserved and lighter shades indicate the presence of base mismatch, base deletion, and base insertion.

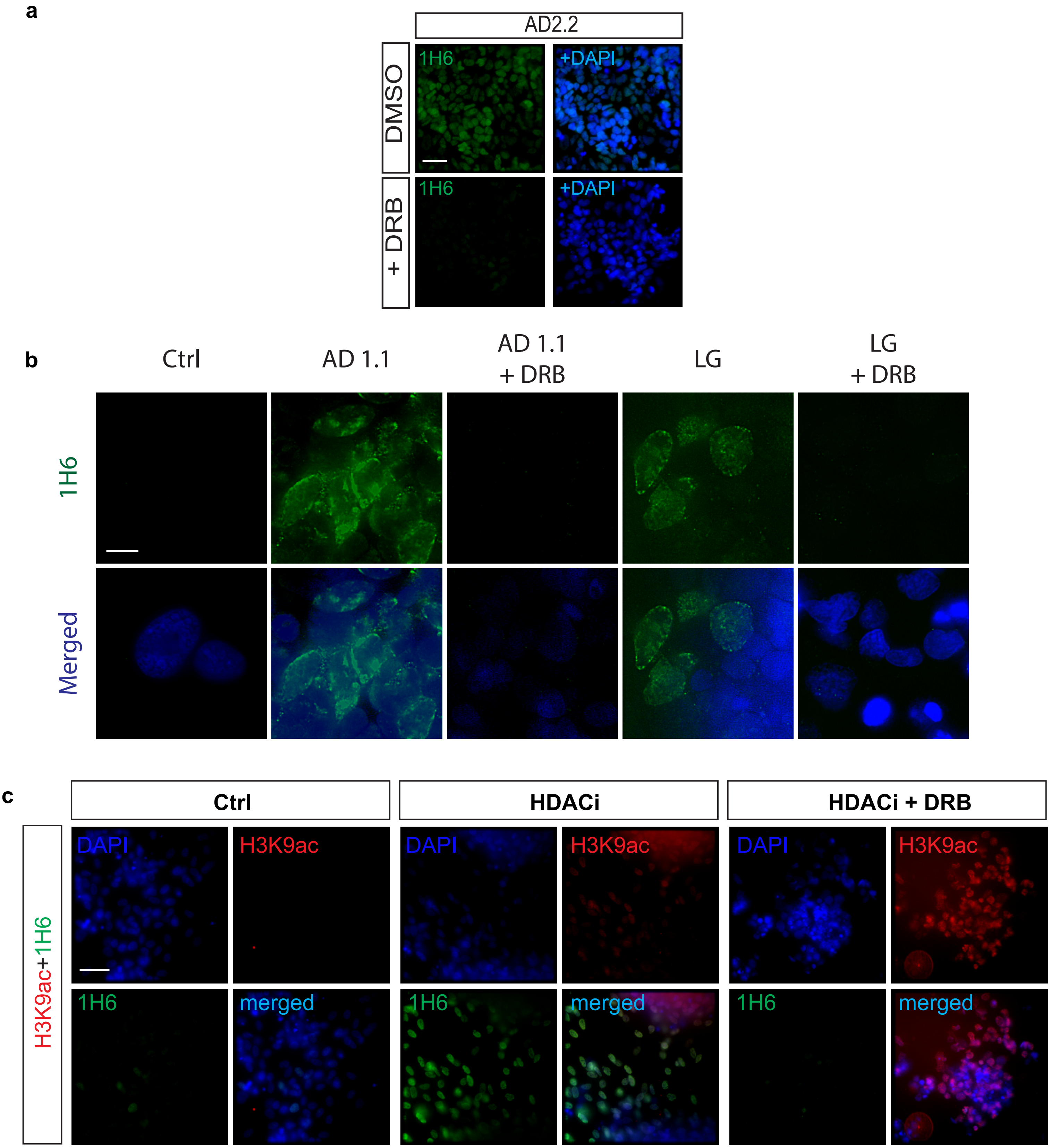

Supplementary Figure 10

Supplementary Figure 10. Inhibition of transcription can rescue the G4 structure phenotype in human neurons

a. AD2 neurons treated for 24h with DRB then fixed with formaldehyde. These cells were immunolabeled with 1H6, counterstained with DAPI. Scale bar: 35 µm.

b. Ctrl or AD neurons treated for 24h with DRB then fixed with formaldehyde. These cells were immunolabeled with 1H6, counterstained with DAPI. Scale bar: 5 µm.

c. Ctrl neurons were treated with HDACi or HDAci and DRB, then fixed with formaldehyde. These cells were immunolabeled with $H3K9^{ac}$ and 1H6, counterstained with DAPI. Scale bar: 35 µm.

a

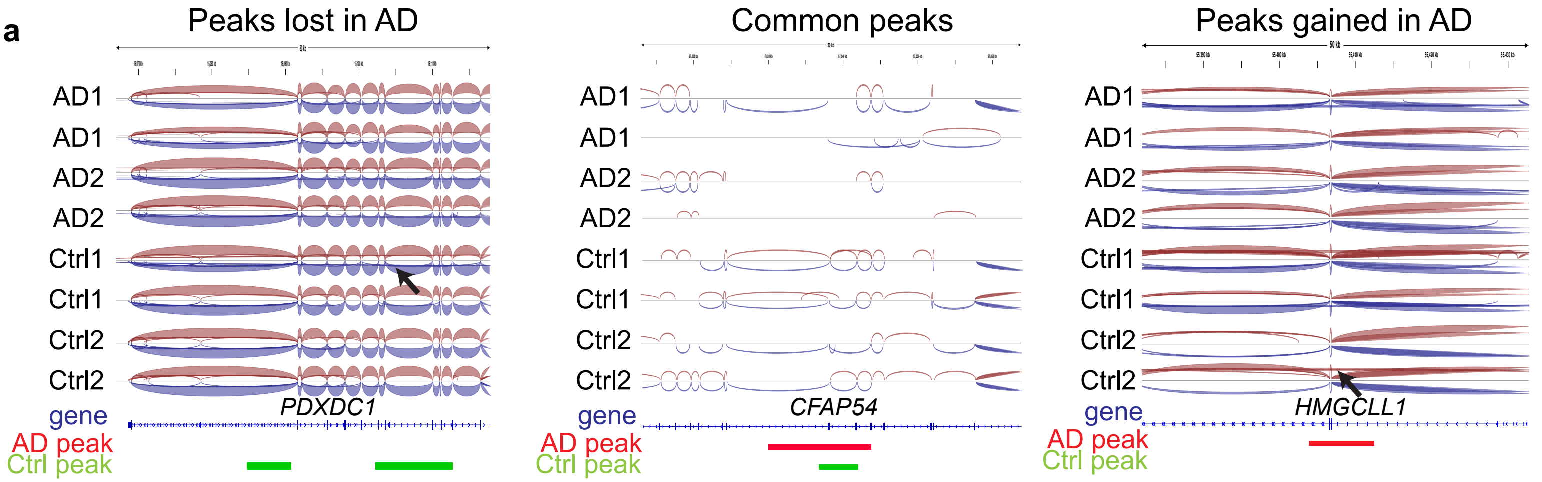

b

	lost in AD	common peaks	gained in AD
genes with peaks	139	259	269
genes with splice variant in AD2	4	15	13
% of splice variants in AD2	2.87	5.8	4.8
minor sv exclusion in AD2	0	2	1
minor sv inclusion in AD2	0	0	0
exon exclusion in AD2	0	12	12
exon inclusion in AD2	4	1	0

c

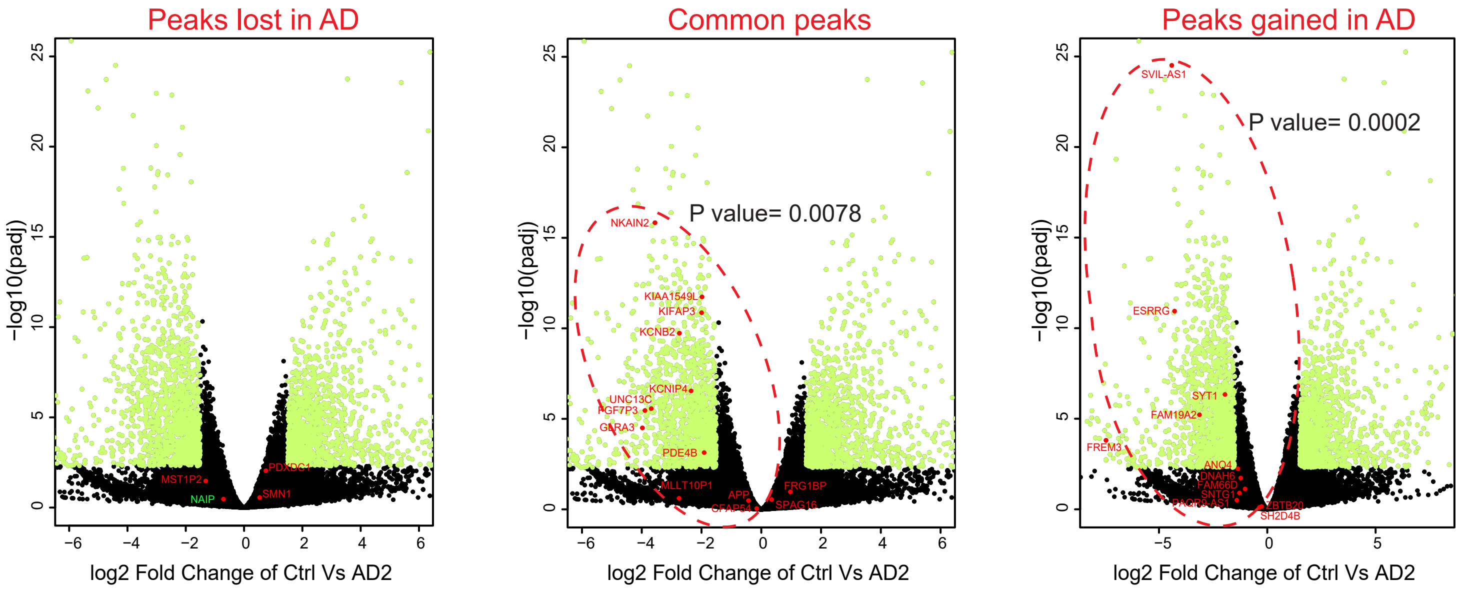

Supplementary Figure 11

Supplementary Figure 11. G4 structures can perturb gene splicing events in human neurons

a. Sashimi plot of three different genes containing a 1H6 peak and having a differential splicing event. The blue line represents the genes with the exons, followed by the lines that represent the 1H6 peak (red: AD neurons; green: Ctrl neurons). The black arrows shows the minor splice variant that is differentially expressed.

b. Table showing the number of genes containing a 1H6 peak, as well as the number of genes that entailed a differential splicing event associated with a 1H6 peak in AD2 when compared to both Ctrls. The splicing events were subdivided into loss of a minor isoform or formation of a gap junction.

c. Volcano plot showing differential gene expression between control (Ctrl1 and Ctrl2 combined) vs AD2 neurons using RNA-seq. Superimposed in red are genes containing a G4 peak and associated with an abnormal splice event. Note that genes in the second and third volcano plot (i.e. common and gained in AD) are significantly downregulated. For each gene distribution (red dotted ovales), a P-value was calculated using binomial distribution, knowing that 62% of the genes were upregulated.

Hanna et al. 2020

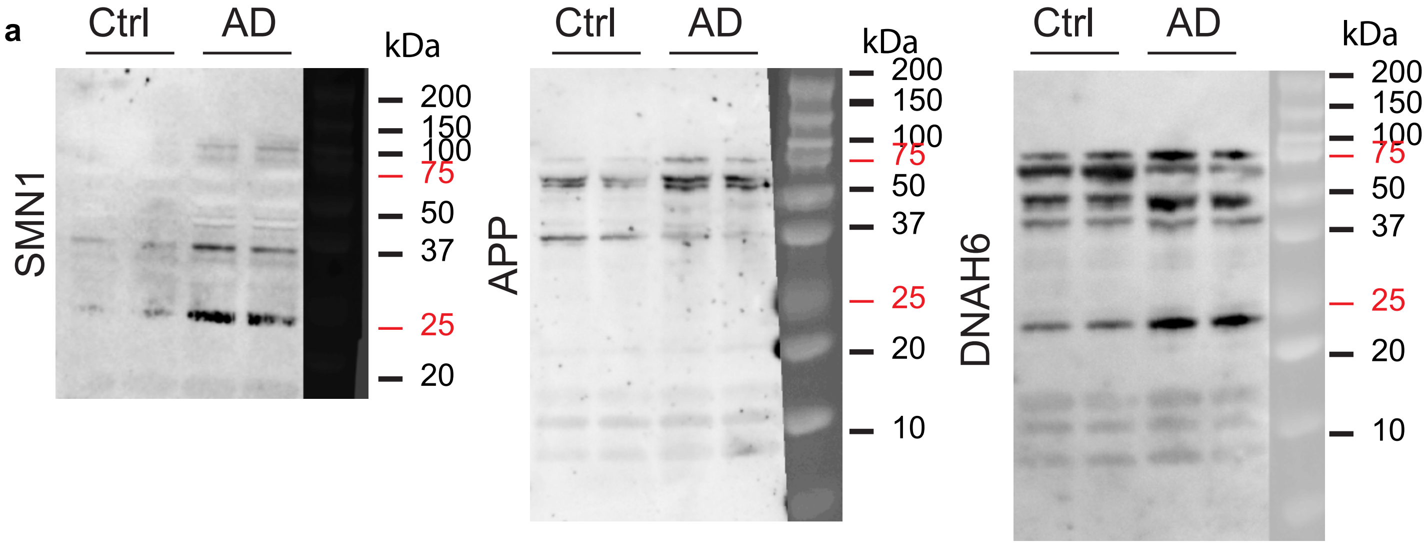

Supplementary Figure 12

Supplementary Figure 12. Full blots of the results presented in Figure 5d

a. Original blots from the analysis of Ctrl2 and AD2 neurons presented in Figure 5d. The protein ladder was added to reveal the molecular weight.

Hanna et al. 2020

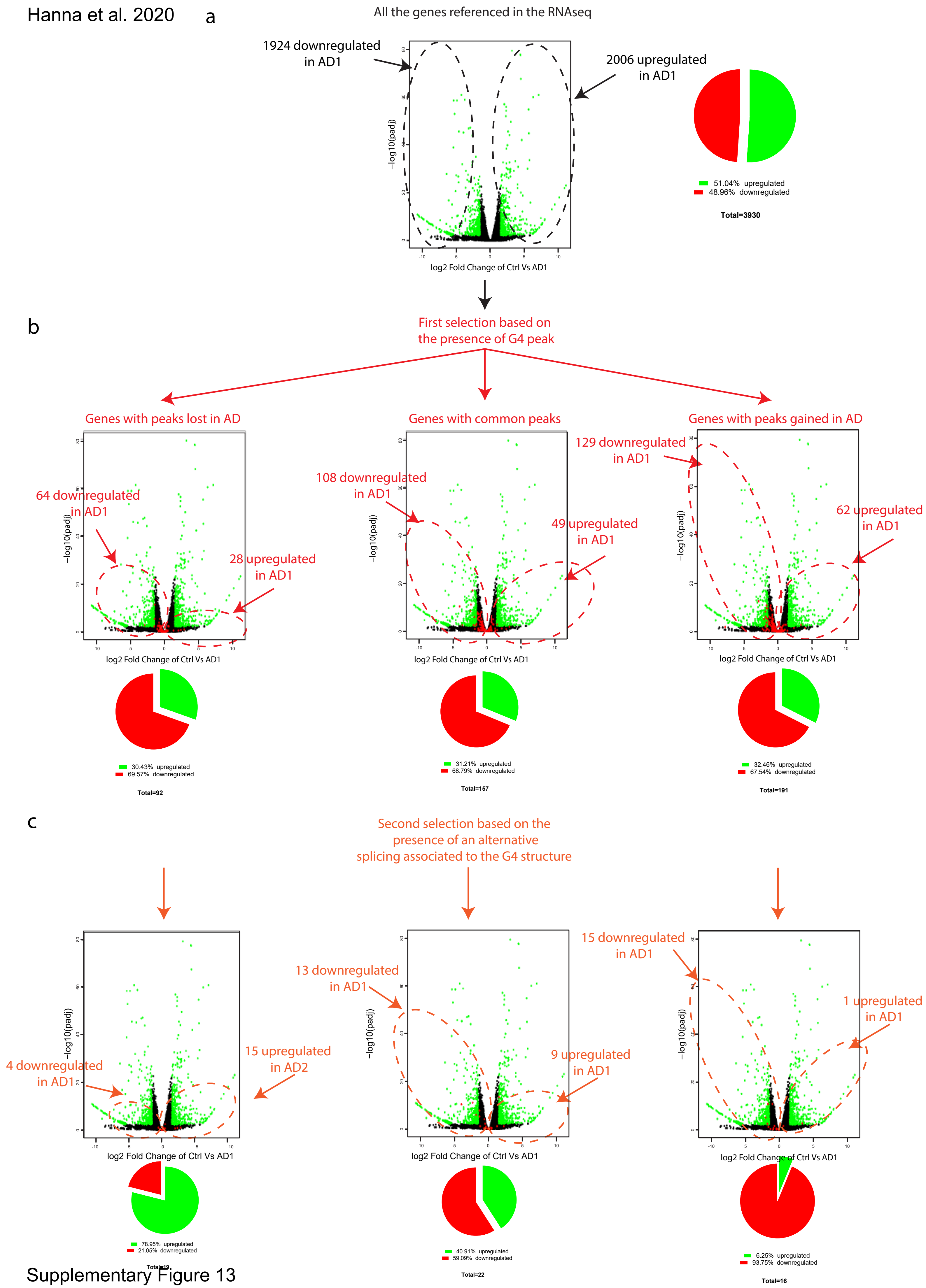

Supplementary Figure 13

Supplementary Figure 13. A step by step walkthrough of the process to study the gene expression profile of AD1 neurons

a. Volcano plot showing the gene expression profile of AD1 compared to control samples (Ctrl1 and Ctrl2 combined). Differentially expressed genes are highlighted in green. A pie chart shows the percentage of upregulated and downregulated genes.

b. The volcano plot from S13a is reproduced and the genes that had a 1H6 peak identified in the ChIP-seq were highlighted and colored in red. The three volcano plots represent the genes that lost a peak in AD, the genes with common peaks, and the genes with a peak gained in AD. The pie charts indicate the percentage of upregulated and downregulated genes within this selection. For example we can notice that the percentage of upregulated genes associated with a peak gained in AD1 went down from 51% in the whole gene pool to 32.4% in the genes affected by a G4 structure. This result indicates that the G4 structures had a negative effect on the expression of the genes where it was located.

c. From the genes identified in S13b we isolated only the genes that had an alternative splicing event linked to a G4 structure. This subselection is now highlighted. The pie charts indicate the percentage of upregulated and downregulated genes within this subselection.

Hanna et al. 2020

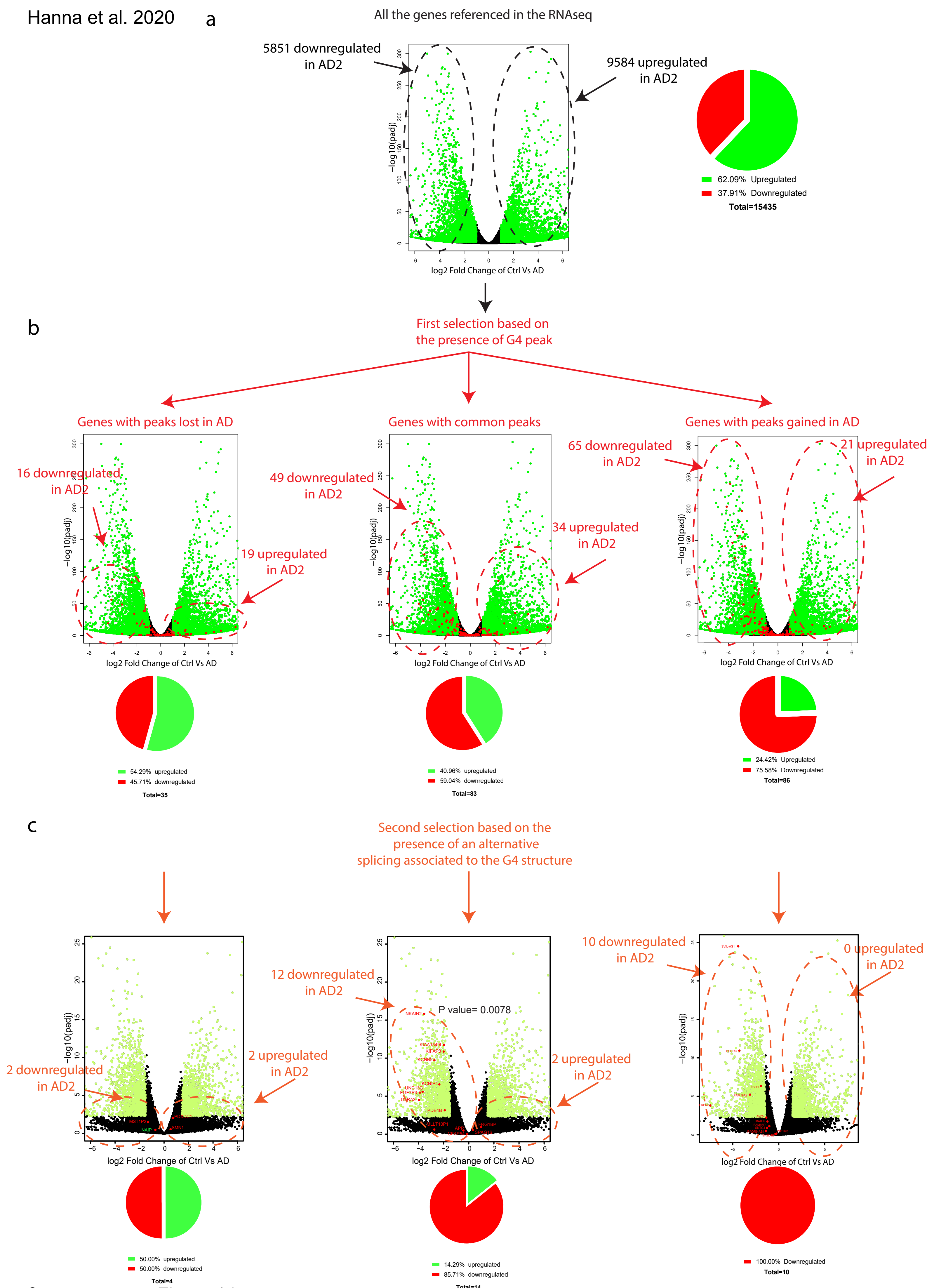

Supplementary Figure 14

Supplementary Figure 14. A step by step walkthrough of the process to study the gene expression profile of AD2 neurons

a. Volcano plot showing the gene expression profile of AD2 compared to control samples (Ctrl1 and Ctrl2 combined). Differentially expressed genes are highlighted in green. A pie chart shows the percentage of upregulated and downregulated genes. We noticed that around 62% of the genes were upregulated in AD2 something to be expected since in AD there is a loss of chromatin compaction and derepression of gene expression.

b. The volcano plot from S14a is reproduced and the genes that had a 1H6 peak identified in the ChIP-seq were highlighted and colored in red. The three volcano plots represent the genes that lost a peak in AD, the genes with common peaks, and the genes with a peak gained in AD. The pie charts indicate the percentage of upregulated and downregulated genes within this selection. For example, we can notice that the percentage of upregulated genes associated with a peak gained in AD went down from 62% in the whole gene pool to 24.4% in the genes affected by a G4 structure. This result indicates that the G4 structures had a negative effect on the expression of the genes where it was located.

c. From the genes identified in S14b we isolated only the genes that had an alternative splicing event linked to a G4 structure. This subselection is now highlighted. The pie charts indicate the percentage of upregulated and downregulated genes within this subselection.
