# Supplementary material for: G-quadruplexes originating from evolutionary conserved L1 elements interfere with neuronal gene expression in Alzheimer’s disease
Source: Nat Commun. 2021 Mar 23;12:1828. doi: 10.1038/s41467-021-22129-9 (PMC7987966; doi:10.1038/s41467-021-22129-9)
Supplement: Supplementary file 3 — Reporting Summary [file 41467_2021_22129_MOESM3_ESM.pdf]

nature research

Corresponding author(s): Dr. Gilbert Bernier

Last updated by author(s): Jan 19, 2021

Reporting Summary

Nature Research wishes to improve the reproducibility of the work that we publish. This form provides structure for consistency and transparency in reporting. For further information on Nature Research policies, see Authors & Referees and the Editorial Policy Checklist.

Statistics

For all statistical analyses, confirm that the following items are present in the figure legend, table legend, main text, or Methods section.

n/a	Confirmed	
☐	☒	The exact sample size (*n*) for each experimental group/condition, given as a discrete number and unit of measurement
☐	☒	A statement on whether measurements were taken from distinct samples or whether the same sample was measured repeatedly
☐	☒	The statistical test(s) used AND whether they are one- or two-sided<br>*Only common tests should be described solely by name; describe more complex techniques in the Methods section.*
☒	☐	A description of all covariates tested
☒	☐	A description of any assumptions or corrections, such as tests of normality and adjustment for multiple comparisons
☐	☒	A full description of the statistical parameters including central tendency (e.g. means) or other basic estimates (e.g. regression coefficient) AND variation (e.g. standard deviation) or associated estimates of uncertainty (e.g. confidence intervals)
☐	☒	For null hypothesis testing, the test statistic (e.g. *F*, *t*, *r*) with confidence intervals, effect sizes, degrees of freedom and *P* value noted<br>*Give P values as exact values whenever suitable.*
☒	☐	For Bayesian analysis, information on the choice of priors and Markov chain Monte Carlo settings
☒	☐	For hierarchical and complex designs, identification of the appropriate level for tests and full reporting of outcomes
☐	☒	Estimates of effect sizes (e.g. Cohen's *d*, Pearson's *r*), indicating how they were calculated

Our web collection on statistics for biologists contains articles on many of the points above.

Software and code

Policy information about availability of computer code

Data collection	ChIP-seq public data were collected using Galaxy platform, and visualized using SeqMonk v0.34.0 software.
Data analysis	Raw reads were aligned on the human genome Hg19 using the torrent platform. EaSeq software (http://easeq.net) (V. 1.111) was used in order to analysis the aligned data, and to call the peaks. EaSeq was also used in order to produce the heatmaps and the train plots with a window of 10 Kbp. Annotation and the statistical significance were done using the RegionR package (V. 1.18.1) using databases downloaded from UCSC table browser. In order to identify the motifs we used the MEME-ChIP from the MEME suite (V.5.0.3). In order to identify the pathways of the identified genes we used Gene Ontology (GO) (V. Panther 14.0).To predict the location of putative G4 sequence we used the Quadparser algorithm (V. 2 running under python V. 2.7.11). Furthermore the seqMINER (V. 8) was used to produce the enrichment heatmaps of H3K9me3. Confocal microscopy analyses were performed using 60x objectives with an IX81 confocal microscope (Olympus, Richmond Hill, Canada), and images were obtained with Fluoview software version 3.1 (FV10-ASW V. 3.1) (Olympus). For the colocalization study, random lines were drawn on individual cells using FIJI (ImageJ 1.52p running under Java 1.8.0_172). For all statistical analysis we used GraphPad Prism V. 5. No custom code was used, or created for this article.

For manuscripts utilizing custom algorithms or software that are central to the research but not yet described in published literature, software must be made available to editors/reviewers. We strongly encourage code deposition in a community repository (e.g. GitHub). See the Nature Research guidelines for submitting code & software for further information.

Data

Policy information about availability of data

All manuscripts must include a data availability statement. This statement should provide the following information, where applicable:

- Accession codes, unique identifiers, or web links for publicly available datasets
- A list of figures that have associated raw data
- A description of any restrictions on data availability

Public data accession numbers used in this study are: GSE22162, GSE44849, GSE76688, GSE38273. The ChIP-seq data of the 1H6 antibody performed in this

publication have been deposited in NCBI's Gene Expression Omnibus and are accessible through GEO Series accession number GSE133113. these data have been used to construct figures 5, 6, and 7 as well as supplementary figures 5, 6 and 7. The RNA-seq data were deposited and are accessible through GEO series accession number GSE162873.

Field-specific reporting

Please select the one below that is the best fit for your research. If you are not sure, read the appropriate sections before making your selection.

☒ Life sciences ☐ Behavioural & social sciences ☐ Ecological, evolutionary & environmental sciences

For a reference copy of the document with all sections, see nature.com/documents/nr-reporting-summary-flat.pdf

Life sciences study design

All studies must disclose on these points even when the disclosure is negative.

Sample size	For iPSCs we used n=3 to compare multiple patient derived cell lines with the controls in order to remove any patient specific biases.
Data exclusions	No data was excluded from the analyses.
Replication	all live cell experiments have been done at least times with reproducible results.
Randomization	the samples were allocated into their respective experimental groups based on their diagnostic provided by Coriell Institute and the NIA aging cell repository. two groups were identified: sporadic Alzheimer disease patients and control samples.
Blinding	during data collection the investigators were blinded to the group allocations. After the data collection and the analysis the group were revealed and data was allocated accordingly.

Reporting for specific materials, systems and methods

We require information from authors about some types of materials, experimental systems and methods used in many studies. Here, indicate whether each material, system or method listed is relevant to your study. If you are not sure if a list item applies to your research, read the appropriate section before selecting a response.

Materials & experimental systems

n/a	Involved in the study	
☐	☒	Antibodies
☐	☒	Eukaryotic cell lines
☒	☐	Palaeontology
☐	☒	Animals and other organisms
☐	☒	Human research participants
☒	☐	Clinical data

Methods

n/a	Involved in the study	
☐	☒	ChIP-seq
☒	☐	Flow cytometry
☒	☐	MRI-based neuroimaging

Antibodies

Antibodies used	Primary antibodies used in this study are: FITC mouse anti-TRA-1-60 (BD Pharmingen, 560380), rabbit anti-SOX2 (ab97959), goat anti-NANOG (R&D systems, af1997), goat anti-S-Opsin (1:250, Santa Cruz, sc-14363), rabbit anti-H3K9me3 (1:500, Abcam, ab8898), rabbit anti-H3K9ac (1:500, Cell Signaling, 9671S), rabbit anti-WRN (1:100, Santa Cruz, sc-5629), rabbit anti-TFIIH p80 (1:200, Santa Cruz, sc-20696) targeted against XPD, rabbit anti-TFIIH p89 (1:200, Santa Cruz, sc-293) targeted against XPB, rabbit anti-53BP1 (1:100, Novus, NB100-304), rabbit anti-H2Aub (1:200, Cell Signaling, 8240S), rabbit anti-SOX2 (1:500, abcam, ab97959), rabbit anti-Ki67 (1:1000, Abcam, ab15580), mouse anti SMN (1:1000, Santa Cruz, SC-32313), rabbit anti-MOAB (1:1000, Novus, NBP2-13075), mouse anti B4 (1:1000, Santa Cruz, SC-28365), rabbit anti ANOA4 (1:1000, Invitrogen, PA5-62785), and rabbit anti DNAO6 (1:1000, Invitrogen, PA5-57636), and mouse BG4 (1:333, Absolute antibody, Ab00174-1.1) and 1H6 antibodies recognizing G-quadruplexes. We obtained the 1H6 antibody from The European Research Institute for the Biology of Ageing. The Secondary antibodies are: donkey AlexaFluor488-conjugated anti-mouse (1:1000, Life Technologies, A21202), donkey AlexaFluor488-conjugated anti-rabbit (1:1000, Life Technologies, A21206), Donkey AlexaFluor647-conjugated anti-mouse (1:1000, Life Technologies, A31571), goat AlexaFluor texas red-conjugated anti-rabbit (1:1000, Life Technologies, T2767).
Validation	A series of validation steps have been done to validate the 1H6 antibody, all these steps are described and explained in the manuscript. in brief, we compared the signal of the 1H6 antibody to that of the BG4 (another G4 structure antibody) and to the WRN signal (a helicase known to resolve the G4 structures) in order to confirm that the localization of these signal is colocalizing. furthermore, we tested if the signal of the 1H6 antibody can be explained solely by the chromatin openness rather than a biological process and found that this was not the case. to add to these factors we also studied and compared the best preparation techniques in order to conserve a specific staining. Finally, the ChIP-seq data provided the final proof that this

antibody was specific. all other antibodies are commercial antibodies that have been validated by their respective manufacturer's and have a track records of citations and publications. as a further step, when we used the antibodies in WB we validated that the band is at the right MW, and when used in IF we validated that the localization of the signal was coherent.

Eukaryotic cell lines

Policy information about cell lines

Cell line source(s)	Normal human diploid fibroblasts (HDFs) were purchased from Coriell Institute. HCA2 cells were kindly provided by the laboratory of Dr. Francis Rodier.
Authentication	The cell lines were not authenticated.
Mycoplasma contamination	all these cells tested negative for mycoplasma contamination on a routine base.
Commonly misidentified lines (See ICLAC register)	No common misidentified cell lines were used in the study.

Animals and other organisms

Policy information about studies involving animals; ARRIVE guidelines recommended for reporting animal research

Laboratory animals	Mice were used in this study, the mice are BMI1 +/- and WT (The Netherlands Cancer Institute, Amsterdam), the background of these mice is C57BL/6J. the mice that were used were equally distributed between male and females and they were sacrifices at P1 and P10 for the respective experiments.
Wild animals	No wild animal was used in this study.
Field-collected samples	No field-collected samples were used in the study.
Ethics oversight	Mice were used in accordance with the Animal Care Committee of the Maisonneuve-Rosemont Hospital Research Centre (Approval ID #2014-03, #2012-09).

Note that full information on the approval of the study protocol must also be provided in the manuscript.

Human research participants

Policy information about studies involving human research participants

Population characteristics	Human brain tissues were obtained from the Banner Brain Banks, two categories were sought: control aged patients that do not represent any sign of Alzheimer disease, and Alzheimer diseased patient that present clear signs of the disease. the age of the samples was between 71 and 88 years old equality distributed between male and females.
Recruitment	Participants were recruited by the Banner Brain Banks. the investigators of this study requested a collaboration with the bank for samples of sporadic Alzheimer disease brains and control aged matched. After the study of the clinical charts these samples were provided for the researchers without any prior biases exerted by the later.
Ethics oversight	Human brain tissues were obtained from the Banner Brain Banks after approval by the Maisonneuve-Rosemont Hospital Ethic Committee.

Note that full information on the approval of the study protocol must also be provided in the manuscript.

ChIP-seq

Data deposition

☒ Confirm that both raw and final processed data have been deposited in a public database such as GEO.

☒ Confirm that you have deposited or provided access to graph files (e.g. BED files) for the called peaks.

Data access links<br>*May remain private before publication.*	https://www.ncbi.nlm.nih.gov/geo/query/acc.cgi?acc=GSE133113, https://www.ncbi.nlm.nih.gov/geo/query/acc.cgi?acc=GSE162873
Files in database submission	Ctrl_1H6-6.IonXpress_RawData.fastq<br>sAD_1H6-6.IonXpress_RawData.fastq<br>Ctrl_input-6.IonXpress_RawData.fastq<br>sAD_input-6.IonXpress_RawData.fastq<br>Ctrl_1H6_enrichment.wig<br>sAD_1H6_enrichment.wig<br>Peaks from 1H6 sAD using imput sAD as negative control.txt<br>Peaks from 1H6 ctrl using imput ctrl as negative control.txt<br>HG1931733.txt<br>Ctrl_1H6_peaks_bedgraph.bed

	sAD_1H6_peaks_bedgraph.bed<br>HG1931733_bedgraph.bed<br>AD1 Vs Ctrl.csv<br>AD2 Vs Ctrl.csv<br>readcount AD1.txt<br>readcount AD2.txt<br>AD11_1.bam<br>AD11_1.bam.bai<br>AD11_2.bam<br>AD11_2.bam.bai<br>A2_P30_1.bam<br>A2_P30_1.bam.bai<br>A2_P30_2.bam<br>A2_P30_2.bam.bai<br>G1_P30_1.bam<br>G1_P30_1.bam.bai<br>G1_P30_2.bam<br>G1_P30_2.bam.bai<br>G2_P30_1.bam<br>G2_P30_1.bam.bai<br>G2_P30_2.bam<br>G2_P30_2.bam.bai<br>HG1931733_bedgraph.bed
Genome browser session (e.g. UCSC)	GSE133113, GSE162873

Methodology

Replicates	Total RNA from two independent biological samples were extracted from each of the two cell line derived from Alzheimer disease patients along with the two cell lines derived from healthy patients for a total of 8 samples.
Sequencing depth	Ctrl_1H6-6.IonXpress_RawData.fastq: 20,165,637 single-end reads of 172 bp<br>sAD_1H6-6.IonXpress_RawData.fastq: 34,270,243 single-end reads of 178 bp<br>Ctrl_input-6.IonXpress_RawData.fastq: 26,031,562 single-end reads of 176 bp<br>sAD_input-6.IonXpress_RawData.fastq: 26,496,348 single-end reads of 175 bp
Antibodies	the 1H6 antibodies recognizing G-quadruplexes was obtained from The European Research Institute for the Biology of Ageing.
Peak calling parameters	read mapping was done using the ion-torrent suite 5.10.1 default settings, the peak calling was done using the Easeq software defaults settings: Ctrl_1H6 raw data were compared to Ctrl_input data and the sAD_1H6 data were compared to sAD_input reads.
Data quality	Peaks were probed with a variable windowsize auto-detected by the software, with p value lower then 1E-5, and a false discovery rate of 1E-5.
Software	the software used in order to call the peaks was EaSeq. (http://easeq.net). For statistical analysis, we used RegionR package on R.
